# Survival Prediction and Feature Selection in Patients with Breast Cancer Using Support Vector Regression

**DOI:** 10.1155/2016/2157984

**Published:** 2016-11-01

**Authors:** Shahrbanoo Goli, Hossein Mahjub, Javad Faradmal, Hoda Mashayekhi, Ali-Reza Soltanian

**Affiliations:** ^1^Department of Biostatistics and Epidemiology, School of Public Health, Hamadan University of Medical Sciences, Hamadan, Iran; ^2^Research Center for Health Sciences, Department of Biostatistics and Epidemiology, School of Public Health, Hamadan University of Medical Sciences, Hamadan, Iran; ^3^Modeling of Non-Communicable Diseases Research Center, Department of Biostatistics and Epidemiology, School of Public Health, Hamadan University of Medical Sciences, Hamadan, Iran; ^4^Computer and IT Engineering Department, Shahrood University, Shahrood, Iran

## Abstract

The Support Vector Regression (SVR) model has been broadly used for response prediction. However, few researchers have used SVR for survival analysis. In this study, a new SVR model is proposed and SVR with different kernels and the traditional Cox model are trained. The models are compared based on different performance measures. We also select the best subset of features using three feature selection methods: combination of SVR and statistical tests, univariate feature selection based on concordance index, and recursive feature elimination. The evaluations are performed using available medical datasets and also a Breast Cancer (BC) dataset consisting of 573 patients who visited the Oncology Clinic of Hamadan province in Iran. Results show that, for the BC dataset, survival time can be predicted more accurately by linear SVR than nonlinear SVR. Based on the three feature selection methods, metastasis status, progesterone receptor status, and human epidermal growth factor receptor 2 status are the best features associated to survival. Also, according to the obtained results, performance of linear and nonlinear kernels is comparable. The proposed SVR model performs similar to or slightly better than other models. Also, SVR performs similar to or better than Cox when all features are included in model.

## 1. Introduction

In the literature, different models have been proposed for survival prediction, such as Cox proportional hazard model and the accelerated failure-time model [1]. Although Cox proportional hazards model is the most popular survival model, its proportional hazards assumption may not always be realistic. Also, this model is applicable when the factors affect the outcome linearly. To overcome these issues, other models such as artificial neural networks and Support Vector Machines (SVM) are applied in the literature [2, 3].

The Support Vector Regression (SVR) model has been applied extensively for response prediction [4–6]. However, there are few studies that use SVM for survival analysis. The traditional SVM models require complete response values, whereas survival data usually include the censored response values. Different attempts have been made to extend the standard SVM model making it applicable in survival analysis. Shivaswamy et al. [7] proposed a modified SVR algorithm for survival analysis. Ding [8] discussed possible application of SVR for censored data. Van Belle et al. [3, 9] proposed a new SVR model using ranking and regression constraints. Another proposal regarding application of SVR in survival analysis was made by Khan and Bayer-Zubek [10]. The authors compared SVR with Cox for some clinical data sets. Van Belle et al. [11] proposed a new survival modeling technique based on least-squares SVR. Their proposed model was compared with different survival models on a clinical BC data set. Mahjub et al. evaluated and compared performance of different SVR models using data sets with different characteristics [12].

SVM does not include automatic feature selection. Feature selection methods for SVM are categorized into two types: filter and wrapper [13]. Wrapper methods are based on SVM, while filter methods are general and not specifically based on SVM.

When dealing with uncensored data, various studies use SVM for feature selection. Many of these studies use feature selection methods for SVM in classification problems [14–18]. For regression problems majority of available approaches propose filter methods [19, 20]. Few studies can be found which propose a wrapper feature selection method for regression [19, 21, 22]. Among these studies is the Recursive Feature Elimination (RFE) method proposed by Guyon et al. [23] for gene selection in cancer diagnosis. Camps-Valls et al. [24] used SVR for prediction and analysis of antisense oligonucleotide efficacy. They applied feature selection using three methods: correlation analysis, mutual information, and SVM-RFE. Becker et al. [18] developed some feature selection methods and used SVM-RFE as a reference model for comparison.

In survival analysis, different feature selection methods are applied in the literature [1, 25–30]. However, using SVR for identifying factors associated with response is rare and to the best of our knowledge, there is no previous study in which SVR is used for evaluating subsets of features. Liu et al. [31] applied SVR with L1 penalty for survival analysis on large datasets. They identified survival-associated prognosis factors for patients with prostate cancer. In costrast to current study, they directly used SVR weights to select the reduced features. Du and Dua [32] discussed the strength of SVR in BC prognosis. They used two feature selection methods neither of which are based on SVR.

The purpose of this study is two-fold. First, we predict the survival time for patients with BC using different SVR models including all available features. We propose a new SVR model using a two-sided loss function for censored data. Performance of Cox model is compared to SVR models by means of three performance measures.

Second, for feature selection, we assess the relationship between every feature and the predicted survival time using relevant statistical tests. We further employ univariate feature selection and the SVM-RFE method to select the best subset of features. Univariate feature selection based on concordance index is proposed in current study. To the best of our knowledge, in the field of survival analysis, there are no previous studies that employ SVM-based feature selection methods. In this paper, all employed feature selection methods are based on SVR.

## 2. Materials and Methods

In this section, first the BC dataset and two available datasets are introduced. Then, different SVR models, Cox regression for censored data, and three feature selection methods are described.

### 2.1. Dataset Description

From April 2004 to April 2011, a historical cohort study was performed on 573 patients with BC, who visited the Oncology Clinic in Institute of Diagnostic and Therapeutic Center orphanage of Hamadan province, in west of Iran. Inclusion criteria were as follows: (i) the patient must be woman, (ii) treated with chemotherapy and radiotherapy, (iii) has undergone one of the related surgery types: breast conservative surgery (lumpectomy), simple mastectomy, radical mastectomy, and partial removal of breast tissue (a quarter or less of the breast) (segmental mastectomy). 31 patients are not eligible and are eliminated from the study. 11 features are selected as independent variables.  Table 1 displays the patients' characteristics. Survival time is defined as the time period from diagnosis to patient death. The patients who survived during the entire study period are considered as right censored. During our study, 201 (37.1%) cancer patients died and 341 (62.9%) patients survived.

Two publicly available datasets (data are available at https://vincentarelbundock.github.io/Rdatasets/datasets.html) are also used for further analysis. The first data set was derived from one of the trials of adjuvant chemotherapy for colon cancer [33]. The dataset includes data of 929 patients. In this experiment (CD), the event under study is recurrence. The variables are as follows: type of treatment, sex, age, time from surgery to registration, extent of local spread, differentiation of tumor, number of lymph nodes, adherence to nearby organs, perforation of colon, and obstruction of colon by tumor. The second data set was obtained from Mayo Clinic trial in primary biliary cirrhosis of the liver [34]. The data set includes 312 randomized participants. There are two events for each person. In a first experiment (PT), the event is transplantation and in a second one (PD) the event is death. There are 17 variables which are as follows: stage, treatment, alkaline phosphatase, edema, age, sex, albumin, bilirubin, cholesterol, aspartate aminotransferase, triglycerides, urine copper, presence of hepatomegaly or enlarged liver, platelet count, standardized blood clotting time, presence of ascites, and blood vessel malformations in the skin.

Some artificial experiments were also conducted to evaluate the effect of number of features included in the model. These experiments are generated with different number of features: 10,20,…, 120. 50 datasets with 200 training and 500 test observations are generated for each feature value. Features values are simulated from a standard normal distribution. Similar methods of dataset generation have been previously applied in the literature [9]. The event time is exponentially distributed with a mean equal to *w* ^*T*^ *x*, where *x* is the feature vector and *w* is the weight vector which is zero for half of features and is generated from a standard normal distribution for the rest of the features [31, 35, 36]. The censoring time is also exponentially distributed with a mean equal to *cw* ^*T*^ *x*, where *c* determines the censoring rate. Other studies applied similar methods of dataset simulation [35, 36].

### 2.2. Standard SVR for Censored Data (Model 1)

Throughout the current text, *x*_*i*_ denotes a *d*-dimensional independent variable vector, *y*_*i*_ is survival time, and *δ*_*i*_ is the censorship status. If an event occurs *δ*_*i*_ is 1, and if the observation is right censored *δ*_*i*_ is 0. In standard SVR, the prognostic index, that is the model prediction, is formulated as

(1) $$\begin{array}{c} u=w^{T}\varphi \left(\;x\;\right)+b, \end{array}$$

where *w* ^*T*^ *φ*(*x*) denotes a linear combination of a transformation of the variables *φ*(*x*) and *b* is a constant. To estimate the prediction, SVR is formulated as an optimization problem and loss function is minimized subject to some constraints [9]. Shivaswamy et al. [7] extended the SVR model for censored data by modifying the constraints of standard SVR. In this paper, the standard SVR model for censored data is named model 1 and formulated as follows.

*Description of the Formulas Used in SVR Models*

 Model 1
  (2) minw,b,ϵ,ϵ∗ 12wTw+γ∑i=1nϵi+ϵi∗,subject  to ∀i=1,…,n, wTφxi+b≥yi−ϵi, −δiwTφxi+b≥−δiyi−ϵi∗, ϵi≥0, ϵi∗≥0.
 Model 2
  (3) minw,b,ϵ,ϵ∗,ξ 12wTw+γ∑i=1nϵi+ϵi∗+μ∑i=1nξi,subject  to ∀i=1,…,n, wTφxi+b≥yi−ϵi, −δiwTφxi+b≥−δiyi−ϵi∗, wTφxi−φxji≥yi−yji−ξi ϵi≥0, ϵi∗≥0, ξi≥0.
 SVR-MRL
  (4) minw,b,ϵ,ϵ∗,ξ 12wTw+γ∑i=1nϵi+ϵi∗+μ∑i=1nξi,subject  to ∀i=1,…,n, wTφxi+b≥yi−ϵi, −δiwTφxi+b≥−δiyi−ϵi∗, δi−1wTφxi+b≥δi−1yi+MRLi−ξi, ϵi≥0, ϵi∗≥0, ξi≥0,

where *γ* and *μ* are positive regularization constants, *ϵ*_*i*_, *ϵ*_*i*_^*∗*^, and *ξ*_*i*_ are slack variables, and *n* is sample size.

The prognostic index for a new point *x* ^*∗*^ is computed as

(5) $$\begin{array}{c} \hat{u}\left(\;x^{*}\;\right)=\underset{i}{\sum }\left(\;\alpha_{i}-\delta_{i}\alpha_{i}^{*}\;\right){\varphi \left(x_{i}\right)}^{T}\varphi \left(\;x^{*}\;\right)+b, \end{array}$$

where *α*_*i*_ and *α*_*i*_^*∗*^ indicate the Lagrange multipliers. *φ*(*x*_*i*_)^*T*^ *φ*(*x*_*j*_) can be formulated as a positive definite kernel:

(6) $$\begin{array}{c} k\left(\;x_{i},x_{j}\;\right)=\varphi {\left(\;x_{i}\;\right)}^{T}\varphi \left(\;x_{j}\;\right). \end{array}$$

Commonly used kernels are linear, polynomial, and RBF [9]. Recently, an additive kernel has been used for clinical data defined as *k*(*x*, *z*) = ∑_*p*=1_^*d*^ *k*_*p*_(*x* ^*p*^, *z* ^*p*^) in which *k*_*p*_(·, ·) is separately defined for continuous and categorical variables [9, 37].

### 2.3. Survival-SVR Using Ranking and Regression Constraints (Model 2)

The SVR model proposed by Van Belle et al. [3, 9] which includes both ranking and regression constraints is called model 2 in this paper and is detailed in “Description of the Formulas Used in SVR Models”. Recall that the previous model (model 1) includes only regression constraints. In model 2, comparable pairs of observations are identified. A data pair is defined to be comparable whenever the order of their event times is known. Data in a pair are comparable if both of them are uncensored, or only one is censored with the censoring time being later than the event time. The number of comparisons is reduced by comparing each observation *i* with its comparable neighbor that has the largest survival time smaller than *y*_*i*_, which is indicated with *y*_*j*(*i*)_ [9].

### 2.4. A New SVR Model Using Mean Residual Lifetime

The survival SVR models, discussed in previous subsections, uses a one-sided loss function for errors arising from prediction of censored observations. We propose a new SVR model which uses a two-sided loss function. This model assumes that the event time for a censored observation is equal to sum of its censoring time and mean residual lifetime (MRL). For individuals of age *y*, MRL measures their expected remaining lifetime using the following formula [1]:

(7) $$\begin{array}{c} MRL\left(\;y\;\right)=\frac{\int_{y}^{\infty }S\left(t\right)dt}{S\left(y\right)}, \end{array}$$

where *S*(*y*) is the survival function. Kaplan–Meier estimator is a standard estimator of the survival function which is used in the current study. Suppose the *i*th individual is censored and *y*_*i*_ shows her/his censoring time. Therefore, it is only known that she/he is alive until *y*_*i*_. Since MRL_*i*_ measures her/his expected remaining lifetime from *y*_*i*_ onwards, event time for this individual can be estimated using sum of censoring time and MRL. Thus, the model is also penalized for censored observations when they are predicted to be greater than this sum. In this paper, this model is called SSVR-MRL and is formulated in “Description of the Formulas Used in SVR Models”.

The parameters of SVR models described in this subsection and previous subsections are tuned using the three-fold cross-validation criterion. We compare different SVR models with the Cox model [1].

The different survival models are compared using three performance measures: concordance index (c-index) [3, 7, 9], log rank test *χ* ^2^ statistic [3, 9, 37], and hazard ratio [3, 9, 10].

Standard SVR (model 1) and SVR with ranking and regression constraints (model 2) based on different kernels are trained on all datasets. In each experiment 2/3 of the dataset is used as the training set and the rest is used as the test set. Each experiment is repeated 100 times with random division of training and testing subsets. The training set is used for estimating different models. The performance of models is evaluated based on the test data.

### 2.5. Feature Selection

The first feature selection method proposed in current study is not a feature subset selection method. Considering that the prognostic index in SVR regression is directly correlated to survival time the prognostic index is calculated for all patients and the relationship between this index and each feature is assessed using a relevant statistical test.

The second feature selection method used in this study is univariate feature selection, which employs a univariate standard linear SVR for evaluating the relationship between each feature and survival time. After implementing experiments for all features, they are arranged in increasing order of c-index. Then, we pick out the p top ranked variables and include them in a linear standard SVR model. In the BC dataset, to select the best value of *p*, both SVR and Cox models are fitted for every value of *p*  (1 − 11), and a subset with highest performance is selected.

The SVM-RFE algorithm, proposed by Guyon et al. [23], is also used for feature selection. This method identifies a subset with size *p* among m features (*p* < *m*) which maximizes the performance measures of model. In this algorithm, the square of feature weight values in linear SVR was used as the ranking criterion [13, 23].

## 3. Results

All reported performance measures are obtained using the median performance on 100 randomizations between training and test sets for prediction and 50 randomizations for feature selection. All SVR models are implemented in Matlab using the Mosek optimization toolbox for Matlab and Yalmip toolbox. Also we use “R”, Version 3.1.2, for running the Cox model and calculating some performance measures.

### 3.1. Prediction of Survival

All measures for the BC dataset indicated that SVR with linear kernel outperformed SVR with nonlinear kernels. The linear SVR models outperformed other models. Using the Wilcoxon rank sum test, statistically significant differences between linear SVR (model 1) and other models are indicated in Table 2. SVR-MRL slightly outperformed linear SVR (model 1), but differences of performances between the two models were not significant.

As the linear standard SVR model significantly outperformed other models, the prognostic indices were obtained based on this model. By comparing the mean value of prognostic index of subgroups of variables, the following results were obtained (Table 1). The survival time decreased as the age increased. The patients who developed metastasis survived less than other patients. The mean of prognostic index in subgroup with smaller tumor size was higher than the mean of subgroup with larger tumor size. The relationship between other variables and survival time can be evaluated similarly.

The results of CD experiment showed that SVR-MRL and SVR with clinical kernel outperformed other models but the difference between their performances and linear SVR (model 1) was not significant. In the PD experiment, SVR-MRL and SVR with polynomial kernel slightly outperformed other models but their performance differences with Linear SVR were not significant. Linear SVR significantly outperformed Cox. In the PT experiment, performances of all models were almost similar except that Cox and SVR with clinical kernel performed significantly worse than linear SVR.

### 3.2. Feature Selection

**Figure 1:**
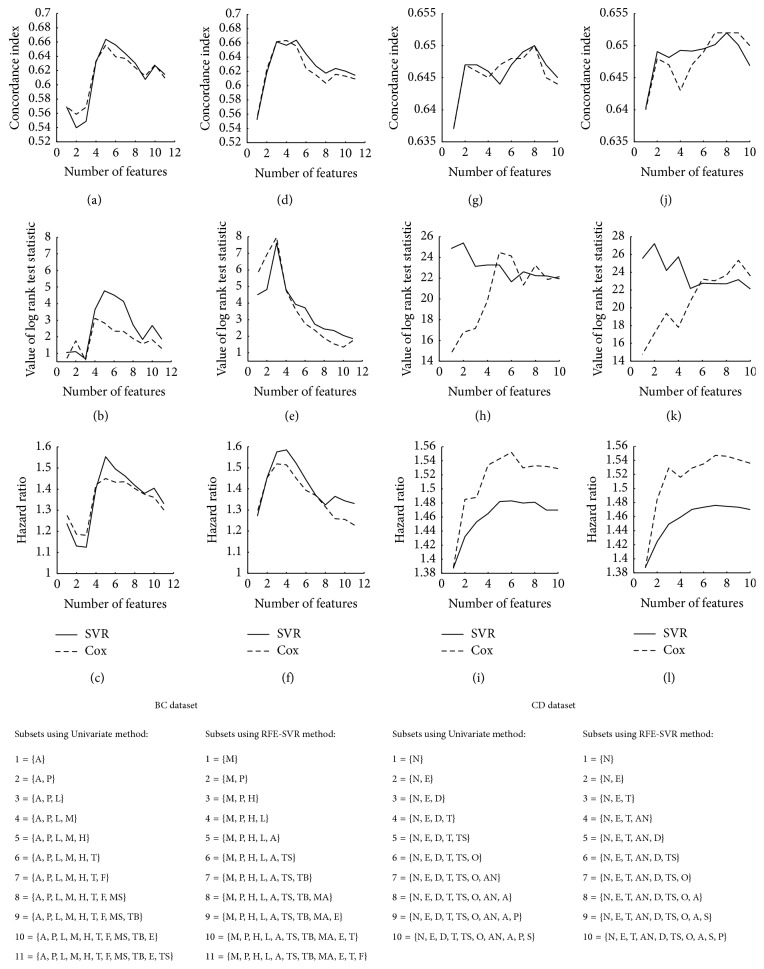
Performance measures of standard linear SVR and Cox for different subsets of features. Horizontal axis shows the number of features included in the model. (a), (b), and (c): performance measures of univariate method for BC dataset. (d), (e), and (f): performance measures of RFE for BC dataset. (g), (h), and (i): performance measures of univariate method for CD dataset. (j), (k), and (l): performance measures of RFE for CD dataset. BC dataset's abbreviations: M: metastasis, P: PR status, H: HER2, L: number of involved lymph nodes, A: age, T: tumor size, TB: histological type of BC, MS: marital status, E: ER status, TS: type of surgery, and F: family history. CD dataset's abbreviations: S: sex, P: perforation of colon, A: age, AN: adherence to nearby organs, O: obstruction of colon by tumor, TS: time from surgery to registration, T: treatment, D: differentiation of tumor, E: extent of local spread, and N: number of lymph nodes.

In the BC dataset, based on the first feature selection method, the relationship between the prognostic index and each feature was evaluated and *p* values of the tests are presented in Table 1. Seven variables were significant (*p* value < 0.05). For univariate feature selection, Figure 1 indicates that the subset with five features has the best performance. In SVR-RFE method, eleven replications were implemented for finding all subset of features. According to the performance measures of subsets, the subset with three features was selected as the best. In this figure, it is clearly shown that when the model involves large number of features, SVR outperforms Cox.

**Figure 2:**
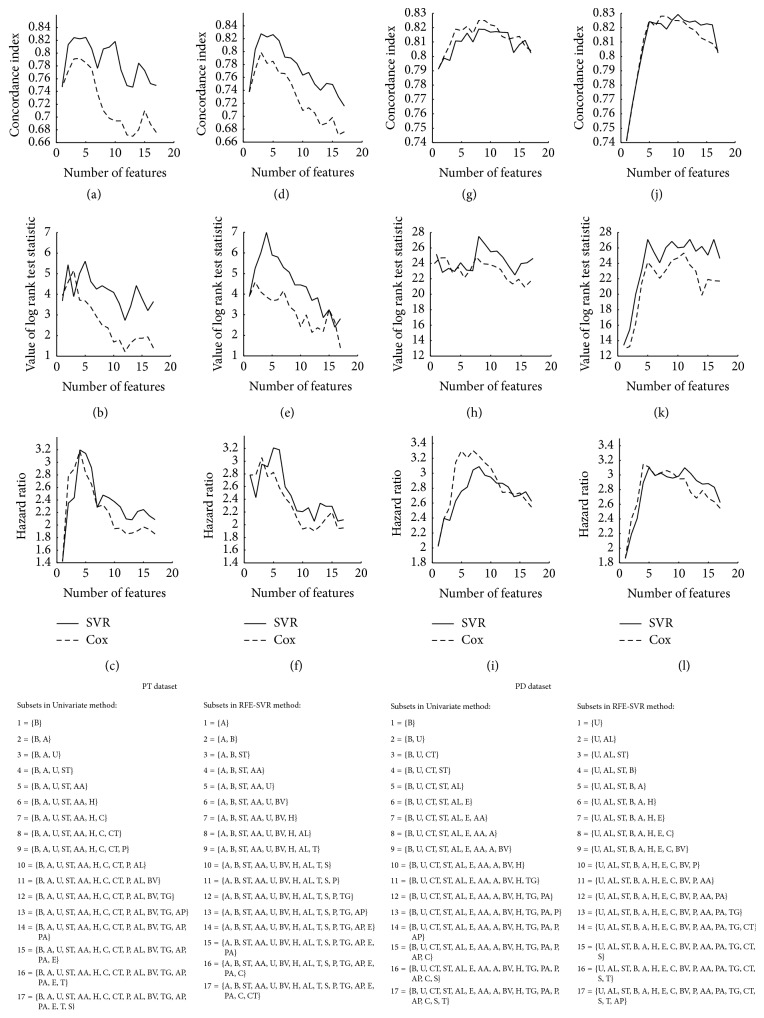
Performance measures of linear SVR and Cox for different subsets of features. (a), (b), and (c): performance measures of univariate method for PT dataset. (d), (e), and (f): performance measures of RFE for PT dataset. (g), (h), and (i): performance measures of univariate method for PD dataset. (j), (k), and (l): performance measures of RFE for PD dataset. B: bilirubin, A: age, U: urine copper, ST: stage, AA: aspartate aminotransferase, H: presence of hepatomegaly, C: cholesterol, CT: standardized blood clotting time, P: platelet count, AL: albumin, BV: blood vessel malformations in the skin, TG: triglycerides, AP: alkaline phosphatase, PA: presence of ascites, E: edema, T: treatment, and S: sex.

In the CD experiment, RFE and univariate methods selected subsets with six and five features respectively, which had five common features (Figure 1). In PT experiment, univariate method selected the subset of five features and RFE method selected the subset of three features among which two features were similar (Figure 2). In the PD experiment, univariate method selected the subset with eight features. RFE selected a subset with five features all of which were observed in the subset selected by RFE. However, in these experiments, the best subset may be not unified. The first feature selection method found most features as significantly associated with the survival time. The selected features had noticeable overlap with two other methods.

It seemed that in subsets with large number of features, SVR outperformed Cox. The results of simulated datasets also indicated similar results so that when the number of features was more than 60, SVR significantly outperformed Cox (Table 3).

## 4. Discussion

The results indicated that, in the BC dataset, linear SVR models outperformed SVR with nonlinear kernel. For other datasets, linear SVR and SVR with nonlinear kernels were comparable. The SVR-MRL model performed similar to or slightly better than other models. This model is based on additivity assumption. The results of this study indicated that SVR model performed similar to or better than Cox when all features were included in model. When feature selection was used, the performance of SVR and Cox was comparable. Although Cox model is based on proportional hazards assumption, an extension of the model was proposed for time-dependent variables when this assumption is not satisfied [1, 38, 39]. However, the additivity of the proposed MRL-method is also a restriction.

Using BC, PT, and PD datasets, the results showed that when the number of included features in model was large, SVR outperformed the Cox model. The results of simulated datasets also indicated that, in models with large number of features, SVR significantly outperformed the Cox model. Du and Dua [32], also using a breast cancer dataset, indicated that SVR performs better than Cox on initial dataset and performs similar to Cox when feature selection is conducted. Van Belle et al. [3, 9] also indicated that the SVR model outperforms the Cox model for high-dimensional data, while for clinical data the models have similar performance.

The experiments indicated that the difference between the simple standard SVR model (model 1) and the complicated SVR model (model 2) was not significant. Using five clinical data sets, Van Belle et al. [9] also found that the differences between two models are not significant.

The results of BC dataset indicated that feature selection methods selected subsets of features with different sizes. However, they were subsets of each other. The three features selected by all methods were as follows: metastasis status, progesterone receptor (PR) status, and human epidermal growth factor receptor 2 (HER2) status. The results indicated that the patients that developed metastasis had shorter life time compared to patients who did not develop metastasis. Some other studies also indicated similar results [25, 27, 40, 41]. Also the results indicated patients with negative HER2 status survived longer than patients with positive HER2 status, which is consistent with results of some previous studies [40, 41]. In addition, the present study yielded that patients with positive PR status had less survival time than the patients with negative PR status. Some other studies indicated contrary result [40, 41] which may be due to the missing values for this variable. However, Faradmal et al. [28] presented similar result for the PR status.

There are other techniques for feature selection. Some studies used statistical characteristics such as correlation coefficients or Fisher score for feature ranking [15, 24, 32]. These criteria are simple, neither being based on training algorithm. In the current study, square of feature weight is used as the ranking criterion which is based on SVR. This criterion was previously applied in other studies [13, 23]. Chang and Lin [15] also ranked each feature by considering how the performance (accuracy) is affected without that. Their method is more time-consuming compared to the feature ranking method used in our study. Also, they found that feature ranking using weights of linear SVR outperforms the feature expelling method in terms of the final model performance. There are some techniques which ranked features based on their influence on the decision hyperplane and were able to also use nonlinear kernels for feature selection [14, 16].

In this study, subset selection methods were used to assess subsets of variables according to their performance in the SVR model. These methods are often criticized due to requiring massive amounts of computation. However, they are robust against overfitting [42]. Also, the better performance of SVM-RFE for feature selection compared with correlation coefficient and mutual information was shown in [23, 24].

Some feature selection methods incorporate variable selection as part of the training using a penalty function [18, 31]. Becker et al. [18] proposed a combination of SCAD and ridge penalties and found it being a robust tool for feature selection. Most of the mentioned techniques were applied for classification problems. It is suggested that, for future studies, these techniques are extended to survival regression models and different feature selection methods are used, evaluated, and compared.

To the best of our knowledge, there is no previous study employing feature selection based on SVR for survival analysis. The univariate method used in this study is a performance-based method which has the advantage of being applicable to all training algorithms. As opposed to this method, SVR-RFE takes into account possible correlation between the variables. For the BC dataset, SVR-RFE method yielded 11 subsets of features one of which was exactly the same as the subset obtained by the first method used in this study. RFE-SVR is an iterative procedure which requires much time for feature selection but the proposed feature selection method is trained once and the results can simultaneously be used for two purposes, prediction, and feature selection. SVR models have some limitations, as well. They require more time for tuning the parameters of the model and the required time for analysis increases with increasing the number of parameters of the model.

## 5. Conclusion

The results for BC data indicated that SVR with linear kernel outperformed SVR with nonlinear kernels. For other datasets, linear SVR and SVR with nonlinear kernels were comparable. Performance of the model improved slightly but not significantly by applying a two-sided loss function. However, the additivity of this model is a restriction.

SVR performed similar or better than Cox when all features were included in model. When feature selection was used, performance of two models was comparable. The result of simulated datasets indicated that when the number of features included in model was large, SVR model significantly outperformed Cox.

Univariate feature selection based on concordance index was proposed in current study which has the advantage of being applicable to all training algorithms. Another method (a combination of SVR and statistical tests) in contrast to univariate and RFE methods is not iterative and can be used for pilot feature selection. For all datasets, the employed methods selected subsets of features with different size, but with high overlaps. Based on the three feature selection methods, metastasis status, progesterone receptor status, and human epidermal growth factor receptor 2 status were the best features associated to survival.

## Figures and Tables

**Table 1:** Comparison of characteristics of dead and alive patients in the BC dataset, and the relation between the features and the estimated prognostic based on standard linear SVR.

Independent variables	Category	Characteristics of patients in dataset				Estimated prognostic index in SVR model		
		Alive		Dead				*p* value
		Count	Mean (SD)	Count	Mean (SD)		Pearson correlation (with prognostic index)	
Age of diagnosis		341	45.33 (10.26)	201	47.30 (11.64)	−0.22		0.001^**∗∗**^
		Count	Percent	Count	Percent	Mean prognostic index	SD prognostic index	
Tumor size (cm)	≤2	230	70.12	98	29.88	−15.98	12.18	0.313
	2–5	103	56.28	80	43.72	−16.47	12.59	
	>5	8	25.80	23	74.2	−23.18	8.12	
Involved lymph nodes (*n*)	<4	198	69.72	86	30.28	−13.44	11.87	<0.001^**∗∗∗**^
	≥4	103	62.05	63	37.95	−21.14	11.32	
	Unknown	40	43.48	52	56.52			
Marital status	Married	326	62.69	194	37.31	−16.20	12.21	0.354
	Single	15	68.18	7	31.82	−20.29	12.93	
Family history	Yes	300	62.11	183	37.89	−16.46	7.12	0.418
	No	41	69.49	18	30.51	−14.94	12.58	
Type of surgery	Lumpectomy	18	64.24	10	35.76	−13.34	9.19	0.031^**∗**^
	Radical mastectomy	250	61.27	158	38.73	−15.47	12.13	
	Segmental mastectomy	26	63.41	15	36.59	−18.64	15.79	
	simple mastectomy	47	72.30	18	27.70	−22.62	9.75	
Histological type	Ductal	301	62.57	180	37.43	−17.18	12.26	0.022^**∗**^
	Lobular	21	61.76	13	38.24	−10.63	10.69	
	Medullar	19	70.37	8	29.63	−10.76	10.81	
Metastases status	Present	51	36.42	89	63.58	−34.32	10.82	<0.001^**∗∗∗**^
	Absent	290	72.13	112	27.87	−12.83	8.99	
HER2	Positive	110	65.47	58	34.53	−20.89	11.62	<0.001^**∗∗∗**^
	Negative	84	60.00	56	40.00	−10.02	10.09	
	Unknown	147	62.82	87	37.18			
ER status	Positive	106	63.85	60	36.15	−17.54	12.35	0.148
	Negative	126	68.10	59	31.90	−15.24	12.06	
	Unknown	109	57.06	82	42.94			
PR status	Positive	85	59.44	58	40.56	−20.39	11.57	<0.001^**∗∗∗**^
	Negative	142	71.71	56	28.29	−13.37	11.88	
	Unknown	114	56.71	87	43.29			

^**∗**^ *p* value < 0.05, ^**∗∗**^ *p* value < 0.01, ^**∗∗∗**^ *p* value < 0.001.

**Table 2:** Performance measures of Cox and SVR models using different kernels and datasets when all features are included in the model. Statistical significant differences between SVR based model 1 (indicated in >italic)  and the other models are indicated based on the Wilcoxon rank sum test.

Dataset	Model	Type of kernel	c-index	Log rank test statistic	Hazard ratio
BC	Cox model	—	0.61 ± 0.03	1.38 ± 1.10	1.22 ± 0.11^*∗∗*^
	*SVR based model 1*	*Linear*	*0.61 ± 0.03*	*1.85 ± 1.48*	*1.33 ± 0.14*
	SVR-MRL based model 1	Linear	0.62 ± 0.03	1.95 ± 1.44	1.33 ± 0.15
	SVR based model 2	Linear	0.60 ± 0.03	1.76 ± 1.33	1.31 ± 0.14
	SVR based model 1	RBF	0.56 ± 0.03^*∗∗∗*^	0.52 ± 0.46^*∗∗∗*^	1.13 ± 0.07^*∗∗∗*^
	SVR based model 2	RBF	0.54 ± 0.05^*∗∗∗*^	0.64 ± 0.64^*∗∗∗*^	1.14 ± 0.11^*∗∗∗*^
	SVR based model 1	Polynomial	0.59 ± 0.04^*∗*^	1.56 ± 1.56	1.23 ± 0.17^*∗∗*^
	SVR based model 2	Polynomial	0.57 ± 0.08^*∗∗∗*^	0.88 ± 0.88^*∗∗∗*^	1.19 ± 0.23^*∗∗∗*^
	SVR based model 1	Clinical	0.60 ± 0.02^*∗*^	1.47 ± 1.11	1.26 ± 0.14
	SVR based model 2	Clinical	0.60 ± 0.04^*∗*^	1.14 ± 0.97^*∗∗*^	1.27 ± 0.14
CD	Cox model	—	0.64 ± 0.01	20.81 ± 5.33	1.53 ± 0.07
	*SVR based model 1*	*Linear*	*0.64 ± 0.01*	*21.59 ± 5.53*	*1.49 ± 0.07*
	SVR based model 2	Linear	0.64 ± 0.01	22.17 ± 6.41	1.51 ± 0.07
	SVR-MRL based model 1	Linear	**0.66 ± 0.01**	**22.60 ± 5.51**	**1.50 ± 0.09**
	SVR based model 1	RBF	0.61 ± 0.01^*∗∗∗*^	12.13 ± 3.49^*∗∗∗*^	1.46 ± 0.08^*∗∗*^
	SVR based model 2	RBF	0.61 ± 0.01^*∗∗∗*^	11.98 ± 3.98^*∗∗∗*^	1.43 ± 0.07^*∗∗∗*^
	SVR based model 1	Polynomial	0.63 ± 0.01^*∗*^	18.94 ± 7.05^*∗*^	1.56 ± 0.11
	SVR based model 2	Polynomial	0.63 ± 0.02^*∗∗*^	14.72 ± 9.65^*∗∗*^	1.54 ± 0.17
	SVR based model 1	Clinical	**0.65 ± 0.01**	**22.63 ± 4.80**	**1.63 ± 0.07**
	SVR based model 2	Clinical	0.65 ± 0.01	22.28 ± 5.12	1.65 ± 0.07
PT	Cox model	—	0.70 ± 0.05^*∗∗*^	2.13 ± 1.50^*∗∗*^	1.41 ± 0.24^*∗∗∗*^
	*SVR based model 1*	*Linear*	*0.74 ± 0.06*	*3.18 ± 2.24*	*2.08 ± 0.49*
	SVR based model 2	Linear	0.74 ± 0.06	3.14 ± 1.92	2.14 ± 0.48
	SVR-MRL based model 1	Linear	**0.76 ± 0.06**	**3.91 ± 2.40**	**2.12 ± 0.49**
	SVR based model 1	RBF	0.68 ± 0.06^*∗∗∗*^	0.81 ± 0.75^*∗∗∗*^	1.45 ± 0.30^*∗∗∗*^
	SVR based model 2	RBF	0.67 ± 0.05^*∗∗∗*^	0.84 ± 0.74^*∗∗∗*^	1.45 ± 0.28^*∗∗∗*^
	SVR based model 1	Polynomial	0.74 ± 0.05	3.69 ± 2.03	2.15 ± 0.52
	SVR based model 2	Polynomial	**0.76 ± 0.06**	**3.76 ± 2.32**	**2.35 ± 0.49**
	SVR based model 1	Clinical	0.71 ± 0.07^*∗∗*^	1.85 ± 1.67^*∗∗*^	1.83 ± 0.46^*∗∗*^
	SVR based model 2	Clinical	0.70 ± 0.07^*∗∗*^	1.60 ± 1.42^*∗∗∗*^	1.83 ± 0.48^*∗∗*^
PD	Cox model	—	0.82 ± 0.02^*∗∗∗*^	23.11 ± 5.24^*∗∗*^	2.67 ± 0.31^*∗∗∗*^
	*SVR based model 1*	*Linear*	*0.84 ± 0.01*	*26.31 ± 5.19*	*3.12 ± 0.55*
	SVR based model 2	Linear	0.83 ± 0.01	27.40 ± 5.59	3.07 ± 0.54
	SVR-MRL based model 1	Linear	0.84 ± 0.01	26.09 ± 5.82	3.14 ± 0.52
	SVR based model 1	RBF	0.84 ± 0.02	27.93 ± 4.46	3.02 ± 0.55
	SVR based model 2	RBF	0.84 ± 0.02	28.51 ± 4.61	3.01 ± 0.59
	SVR based model 1	Polynomial	0.84 ± 0.02	26.58 ± 4.52	3.02 ± 0.52
	SVR based model 2	Polynomial	0.84 ± 0.02	26.61 ± 4.85	3.12 ± 0.49
	SVR based model 1	Clinical	0.83 ± 0.01^*∗∗*^	23.92 ± 4.80^*∗*^	3.21 ± 0.56
	SVR based model 2	Clinical	0.83 ± 0.01^*∗∗*^	25.11 ± 5.23^*∗*^	3.14 ± 0.54

^*∗*^ *p* value < 0.05, ^*∗∗*^ *p* value < 0.01, ^*∗∗∗*^ *p* value < 0.001 (Wilcoxon rank sum test).

**Table 3:** Performance measures of SVR and Cox models for simulated experiments with different number of features.

Number of features	Model	c-index	Log rank test statistic	Hazard ratio
10	SVR	0.562 ± 0.015	12.281 ± 4.955	1.240 ± 0.063
	Cox	0.560 ± 0.013	10.662 ± 5.234	1.245 ± 0.060
20	SVR	0.552 ± 0.013	8.589 ± 5.557	1.203 ± 0.048
	Cox	0.552 ± 0.013	9.289 ± 5.990	1.194 ± 0.054
30	SVR	0.540 ± 0.010	5.070 ± 2.275	1.156 ± 0.042
	Cox	0.541 ± 0.011	5.510 ± 3.907	1.154 ± 0.041
40	SVR	0.535 ± 0.011	4.900 ± 2.907	1.117 ± 0.029
	Cox	0.538 ± 0.011	4.367 ± 2.573	1.125 ± 0.035
50	SVR	0.540 ± 0.014	5.234 ± 3.557	1.136 ± 0.054
	Cox	0.537 ± 0.013	3.874 ± 2.681	1.120 ± 0.047
60	SVR	0.532 ± 0.012	3.474 ± 2.748	1.105 ± 0.039
	Cox	0.528 ± 0.015	3.723 ± 2.662	1.112 ± 0.052
70	SVR	0.535 ± 0.012	4.134 ± 2.304	1.123 ± 0.036
	Cox	0.528 ± 0.016	2.774 ± 1.925^*∗*^	1.096 ± 0.044^*∗*^
80	SVR	0.530 ± 0.010	3.171 ± 2.597	1.120 ± 0.036
	Cox	0.526 ± 0.010^*∗*^	1.687 ± 1.323	1.079 ± 0.041^*∗∗*^
90	SVR	0.526 ± 0.013	2.846 ± 2.219	1.098 ± 0.030
	Cox	0.521 ± 0.012^*∗*^	1.720 ± 1.492^*∗*^	1.070 ± 0.041^*∗∗*^
100	SVR	0.524 ± 0.012	1.594 ± 1.386	1.085 ± 0.045
	Cox	0.517 ± 0.014^*∗∗*^	1.226 ± 1.033	1.054 ± 0.030^*∗*^
110	SVR	0.535 ± 0.012	3.738 ± 3.335	1.101 ± 0.048
	Cox	0.515 ± 0.008^*∗∗∗*^	0.870 ± 0.800^*∗∗∗*^	1.057 ± 0.024^*∗∗∗*^
120	SVR	0.527 ± 0.012	1.863 ± 1.581	1.091 ± 0.035
	Cox	0.515 ± 0.015^*∗∗*^	1.092 ± 0.964^*∗*^	1.058 ± 0.033^*∗∗*^

^*∗*^ *p* value < 0.05, ^*∗∗*^ *p* value < 0.01, ^*∗∗∗*^ *p* value < 0.001 (Wilcoxon rank sum test).
